# Supplementary material for: What determines the ‘culture of silence’? Disclosing and reporting sexual harassment among university employees and students at a large Swedish public university
Source: PLoS One. 2025 Mar 26;20(3):e0319407. doi: 10.1371/journal.pone.0319407 (PMC11942412; doi:10.1371/journal.pone.0319407)
Supplement: S1 Table — (DOCX) [file pone.0319407.s001.docx]

**S1 Table I. Items of sexual harassment behavior in the Lund University Sexual Harassment Inventory (LUSHI).**

Exposure to any of the behaviors listed below was coded as exposure to sexual harassment.

Wording of this question in the survey:

We will now ask some questions about your experiences of sexual harassment and sexual violence. Sexual harassment is defined as conduct of a sexual nature that violates someone’s dignity. This can be, for example, through comments or words, groping or indiscreet looks. It can also include unwelcome compliments, invitations, or suggestive acts. Sexual violence is defined in this study as attempts to conduct, or the conduct of sexual acts in which the person did not participate voluntarily.

Have you experienced any of the following situations during your employment/your time as a student at your university?’

| Unwelcome suggestive looks or gestures * |
| --- |
| Unwelcome ‘inadvertent’ brushing or touching * |
| Unwelcome bodily contact such as grabbing or fondling * |
| Unwelcome comments * |
|  |
| Unwelcome soliciting or pressuring for ‘dates’ ** |
| Unwelcome contact online, for example social media or email ** |
| Unwelcome contact by post or telephone ** |
| Unwelcome gifts ** |
| Stalking ** |
|  |
| Attempted or completed rape *** |

* categorized (1) as sexual harassment of non-soliciting type

** categorized as sexual harassment of soliciting type

*** not categorized into any of these two types

1. Östergren PO, Canivet C, Priebe G, Agardh A. Validation of Lund University Sexual Harassment Inventory (LUSHI)-A Proposed Instrument for Assessing Sexual Harassment among University Employees and Students. Int J Environ Res Public Health. 2022;19(24). DOI 10.3390/ijerph192417085

**Table 6 A-F.**

Association between perpetrator gender and tendency of disclosing/reporting SH, using individuals stratified by gender.
Individuals with missing data are excluded.

Table 6 A. University staff and PhD students (N=454); Disclosed

| Gender of perpetrator or perpetrators  (in one or several  SH events) | Exposed persons | | | | | | | | | | | |
| --- | --- | --- | --- | --- | --- | --- | --- | --- | --- | --- | --- | --- |
|  | Women | | | | Men | | | | Non-binary | | | |
|  | No | | Yes | | No | | Yes | | No | | Yes | |
|  | N | % | N | % | N | % | N | % | N | % | N | % |
| Men only | 208 | 59.8 | 140 | 40.2 | 5 | 62.5 | 3 | 37.5 | 4 | 57.1 | 3 | 42.9 |
| Women only | 2 | 50.0 | 2 | 50.0 | 30 | 60.0 | 20 | 40.0 | 1 | 100.0 | 0 | 0 |
| Unknown only | 9 | 75.0 | 3 | 25.0 | 6 | 66.7 | 3 | 33.3 | - | - | - | - |
| Men + Women | 2 | 28.6 | 5 | 71.4 | 3 | 60.0 | 2 | 40.0 | - | - | - | - |
| Men + Unknown | 0 | 0 | 1 | 100.0 | - | - | - | - | - | - | - | - |
| Men + Women + Unknown | - | - | - | - | 1 | 100.0 | 0 | 0 | - | - | - | - |
| Women + Unknown | - | - | - | - | 1 | 100.0 | 0 | 0 | - | - | - | - |
| *Sum* | *221* |  | *151* |  | *46* |  | *28* |  | *5* |  | *3* |  |

Table 6 B. University staff and PhD students (N=454); Reported

| Gender of perpetrator or perpetrators  (in one or several  SH events) | Exposed persons | | | | | | | | | | | |
| --- | --- | --- | --- | --- | --- | --- | --- | --- | --- | --- | --- | --- |
|  | Women | | | | Men | | | | Non-binary | | | |
|  | No | | Yes | | No | | Yes | | No | | Yes | |
|  | N | % | N | % | N | % | N | % | N | % | N | % |
| Men only | 291 | 83.6 | 57 | 16.4 | 7 | 87.5 | 1 | 12.5 | 5 | 71.4 | 2 | 28.6 |
| Women only | 2 | 50.0 | 2 | 50.0 | 40 | 80.0 | 10 | 20.0 | 1 | 100.0 | 0 | 0 |
| Unknown only | 10 | 83.3 | 2 | 16.7 | 8 | 88.9 | 1 | 11.1 | - | - | - | - |
| Men + Women | 4 | 57.1 | 3 | 42.9 | 3 | 60.0 | 2 | 40.0 | - | - | - | - |
| Men + Unknown | 0 | 0 | 1 | 100.0 | - | - | - | - | - | - | - | - |
| Men + Women + Unknown | - | - | - | - | 1 | 100.0 | 0 | 0 | - | - | - | - |
| Women + Unknown | - | - | - | - | 1 | 100.0 | 0 | 0 | - | - | - | - |
| *Sum* | *307* |  | *65* |  | *60* |  | *14* |  | *6* |  | *2* |  |

Table 6 C. Students (N=1988); Disclosed

| Gender of perpetrator or perpetrators  (in one or several  SH events) | Exposed persons | | | | | | | | | | | |
| --- | --- | --- | --- | --- | --- | --- | --- | --- | --- | --- | --- | --- |
|  | Women | | | | Men | | | | Non-binary | | | |
|  | No | | Yes | | No | | Yes | | No | | Yes | |
|  | N | % | N | % | N | % | N | % | N | % | N | % |
| Men only | 1325 | 88.2 | 177 | 11.8 | 80 | 89.9 | 9 | 10.1 | 14 | 93.3 | 1 | 6.7 |
| Women only | 28 | 93.3 | 2 | 6.7 | 214 | 93.4 | 15 | 6.6 | 1 | 100.0 | 0 | 0 |
| Non-binary only | 1 | 25.0 | 3 | 75.0 | 1 | 50.0 | 1 | 50.0 | - | - | - | - |
| Unknown only | 7 | 87.5 | 1 | 12.5 | 18 | 90.0 | 2 | 10.0 | 1 | 100.0 | 0 | 0 |
| Men + Women | 30 | 81.1 | 7 | 18.9 | 36 | 94.7 | 2 | 5.3 | - | - | - | - |
| Men + Non-binary | 0 | 0 | 1 | 100.0 | - | - | - | - | - | - | - | - |
| Men + Unknown | 4 | 100.0 | 0 | 0 | - | - | - | - | 1 | 100.0 | 0 | 0 |
| Men + Women + Unknown | - | - | - | - | 1 | 100.0 | 0 | 0 |  |  |  |  |
| Men + Women + Non-binary + Unknown | - | - | - | - | - | - | - | - | 1 | 50.0 | 1 | 50.0 |
| Women + Non-binary | - | - | - | - | 2 | 100.0 | 0 | 0 | - | - | - | - |
| Women + Unknown | 1 | 100.0 | 0 | 0 | - | - | - | - | - | - | - | - |
| *Sum* | *1396* |  | *191* |  | *352* |  | *29* |  | *18* |  | *2* |  |

Table 6 D. Students (N=1988); Reported

| Gender of perpetrator or perpetrators  (in one or several  SH events) | Exposed persons | | | | | | | | | | | |
| --- | --- | --- | --- | --- | --- | --- | --- | --- | --- | --- | --- | --- |
|  | Women | | | | Men | | | | Non-binary | | | |
|  | No | | Yes | | No | | Yes | | No | | Yes | |
|  | N | % | N | % | N | % | N | % | N | % | N | % |
| Men only | 1441 | 95.9 | 61 | 4.1 | 87 | 97.8 | 2 | 2.2 | 14 | 93.3 | 1 | 6.7 |
| Women only | 28 | 93.3 | 2 | 6.7 | 225 | 98.3 | 4 | 1.7 | 1 | 100.0 | 0 | 0 |
| Non-binary only | 1 | 25.0 | 3 | 75.0 | 2 | 100.0 | 0 | 0 | - | - | - | - |
| Unknown only | 8 | 100.0 | 0 | 0 | 19 | 95.0 | 1 | 5.0 | 1 | 100.0 | 0 | 0 |
| Men + Women | 36 | 97.3 | 1 | 2.7 | 36 | 94.7 | 2 | 5.3 | - | - | - | - |
| Men + Non-binary | 1 | 100.0 | 0 | 0 | - | - | - | - | - | - | - | - |
| Men + Unknown | 4 | 100.0 | 0 | 0 | - | - | - | - | 1 | 100.0 | 0 | 0 |
| Men + Women + Unknown | - | - | - | - | 1 | 100.0 | 0 | 0 | - | - | - | - |
| Men + Women + Non-binary + Unknown | - | - | - | - | - | - | - | - | 1 | 50.0 | 1 | 50.0 |
| Women + Non-binary | - | - | - | - | 2 | 100.0 | 0 | 0 | - | - | - | - |
| Women + Unknown | 1 | 100.0 | 0 | 0 | - | - | - | - | - | - | - | - |
| *Sum* | *1520* |  | *67* |  | *372* |  | *9* |  | *18* |  | *2* |  |

Table 6 E. University staff and PhD students.
N=410; Women and men who had been exposed by men only or by women only

| Gender of perpetrator or perpetrators  (in one or several  SH events) | Exposed persons | | | | | | | | | |
| --- | --- | --- | --- | --- | --- | --- | --- | --- | --- | --- |
|  | Women | | | | | Men | | | | |
|  | Disclosed | | | | | | | | | |
|  | No | | Yes | |  | No | | Yes | |  |
|  | N | % | N | % | Pearson chi square | N | % | N | % | Pearson chi square |
| Men only | 208 | 59.8 | 140 | 40.2 |  | 5 | 62.5 | 3 | 37.6 |  |
| Women only | 2 | 50.0 | 2 | 50.0 | 0.69 | 30 | 60.0 | 20 | 40.0 | 0.89 |
|  |  |  |  |  |  |  |  |  |  |  |
|  | Reported | | | | | | | | | |
|  | No | | Yes | |  | No | | Yes | |  |
|  | N | % | N | % | Pearson chi square | N | % | N | % | Pearson chi square |
| Men only | 291 | 93.6 | 57 | 16.4 |  | 7 | 87.5 | 1 | 12.5 |  |
| Women only | 2 | 50.0 | 2 | 50.0 | 0.07 | 40 | 80.0 | 10 | 20.0 | 0.62 |

Table F. Students.
N=1850; Women and men who had been exposed by men only or by women only

| Gender of perpetrator or perpetrators  (in one or several  SH events) | Exposed persons | | | | | | | | | |
| --- | --- | --- | --- | --- | --- | --- | --- | --- | --- | --- |
|  | Women | | | | | Men | | | | |
|  | Disclosed | | | | | | | | | |
|  | No | | Yes | |  | No | | Yes | |  |
|  | N | % | N | % | Pearson chi square | N | % | N | % | Pearson chi square |
| Men only | 1325 | 88.2 | 177 | 11.8 |  | 80 | 89.9 | 9 | 10.1 |  |
| Women only | 28 | 93.3 | 2 | 6.7 | 0.39 | 214 | 93.4 | 15 | 6.6 | 0.28 |
|  | | | | | | | | | | |
|  | Reported | | | | | | | | | |
|  | No | | Yes | |  | No | | Yes | |  |
|  | N | % | N | % | Pearson chi square | N | % | N | % | Pearson chi square |
| Men only | 1441 | 95.9 | 61 | 4.1 |  | 87 | 97.8 | 2 | 2.2 |  |
| Women only | 28 | 93.3 | 2 | 6.7 | 0.48 | 225 | 98.3 | 4 | 1.7 | 0.77 |
