# Supplementary material for: What determines the ‘culture of silence’? Disclosing and reporting sexual harassment among university employees and students at a large Swedish public university
Source: PLoS One. 2025 Mar 26;20(3):e0319407. doi: 10.1371/journal.pone.0319407 (PMC11942412; doi:10.1371/journal.pone.0319407)
Supplement: S2 Table — (DOCX) [file pone.0319407.s002.docx]

S2 **Table II A-F.** Association between perpetrator gender and tendency of disclosing/reporting SH, using individuals stratified by gender.

Individuals with missing data are excluded.

Table II A. University staff and PhD students (N=454); Disclosed

| Gender of perpetrator or perpetrators  (in one or several  SH events) | Exposed persons | | | | | | | | | | | |
| --- | --- | --- | --- | --- | --- | --- | --- | --- | --- | --- | --- | --- |
|  | Women | | | | Men | | | | Non-binary | | | |
|  | No | | Yes | | No | | Yes | | No | | Yes | |
|  | N | % | N | % | N | % | N | % | N | % | N | % |
| Men only | 208 | 59.8 | 140 | 40.2 | 5 | 62.5 | 3 | 37.5 | 4 | 57.1 | 3 | 42.9 |
| Women only | 2 | 50.0 | 2 | 50.0 | 30 | 60.0 | 20 | 40.0 | 1 | 100.0 | 0 | 0 |
| Unknown only | 9 | 75.0 | 3 | 25.0 | 6 | 66.7 | 3 | 33.3 | - | - | - | - |
| Men + Women | 2 | 28.6 | 5 | 71.4 | 3 | 60.0 | 2 | 40.0 | - | - | - | - |
| Men + Unknown | 0 | 0 | 1 | 100.0 | - | - | - | - | - | - | - | - |
| Men + Women + Unknown | - | - | - | - | 1 | 100.0 | 0 | 0 | - | - | - | - |
| Women + Unknown | - | - | - | - | 1 | 100.0 | 0 | 0 | - | - | - | - |
| *Sum* | *221* |  | *151* |  | *46* |  | *28* |  | *5* |  | *3* |  |

Table II B. University staff and PhD students (N=454); Reported

| Gender of perpetrator or perpetrators  (in one or several  SH events) | Exposed persons | | | | | | | | | | | |
| --- | --- | --- | --- | --- | --- | --- | --- | --- | --- | --- | --- | --- |
|  | Women | | | | Men | | | | Non-binary | | | |
|  | No | | Yes | | No | | Yes | | No | | Yes | |
|  | N | % | N | % | N | % | N | % | N | % | N | % |
| Men only | 291 | 83.6 | 57 | 16.4 | 7 | 87.5 | 1 | 12.5 | 5 | 71.4 | 2 | 28.6 |
| Women only | 2 | 50.0 | 2 | 50.0 | 40 | 80.0 | 10 | 20.0 | 1 | 100.0 | 0 | 0 |
| Unknown only | 10 | 83.3 | 2 | 16.7 | 8 | 88.9 | 1 | 11.1 | - | - | - | - |
| Men + Women | 4 | 57.1 | 3 | 42.9 | 3 | 60.0 | 2 | 40.0 | - | - | - | - |
| Men + Unknown | 0 | 0 | 1 | 100.0 | - | - | - | - | - | - | - | - |
| Men + Women + Unknown | - | - | - | - | 1 | 100.0 | 0 | 0 | - | - | - | - |
| Women + Unknown | - | - | - | - | 1 | 100.0 | 0 | 0 | - | - | - | - |
| *Sum* | *307* |  | *65* |  | *60* |  | *14* |  | *6* |  | *2* |  |

Table II C. Students (N=1988); Disclosed

| Gender of perpetrator or perpetrators  (in one or several  SH events) | Exposed persons | | | | | | | | | | | |
| --- | --- | --- | --- | --- | --- | --- | --- | --- | --- | --- | --- | --- |
|  | Women | | | | Men | | | | Non-binary | | | |
|  | No | | Yes | | No | | Yes | | No | | Yes | |
|  | N | % | N | % | N | % | N | % | N | % | N | % |
| Men only | 1325 | 88.2 | 177 | 11.8 | 80 | 89.9 | 9 | 10.1 | 14 | 93.3 | 1 | 6.7 |
| Women only | 28 | 93.3 | 2 | 6.7 | 214 | 93.4 | 15 | 6.6 | 1 | 100.0 | 0 | 0 |
| Non-binary only | 1 | 25.0 | 3 | 75.0 | 1 | 50.0 | 1 | 50.0 | - | - | - | - |
| Unknown only | 7 | 87.5 | 1 | 12.5 | 18 | 90.0 | 2 | 10.0 | 1 | 100.0 | 0 | 0 |
| Men + Women | 30 | 81.1 | 7 | 18.9 | 36 | 94.7 | 2 | 5.3 | - | - | - | - |
| Men + Non-binary | 0 | 0 | 1 | 100.0 | - | - | - | - | - | - | - | - |
| Men + Unknown | 4 | 100.0 | 0 | 0 | - | - | - | - | 1 | 100.0 | 0 | 0 |
| Men + Women + Unknown | - | - | - | - | 1 | 100.0 | 0 | 0 |  |  |  |  |
| Men + Women + Non-binary + Unknown | - | - | - | - | - | - | - | - | 1 | 50.0 | 1 | 50.0 |
| Women + Non-binary | - | - | - | - | 2 | 100.0 | 0 | 0 | - | - | - | - |
| Women + Unknown | 1 | 100.0 | 0 | 0 | - | - | - | - | - | - | - | - |
| *Sum* | *1396* |  | *191* |  | *352* |  | *29* |  | *18* |  | *2* |  |

Table II D. Students (N=1988); Reported

| Gender of perpetrator or perpetrators  (in one or several  SH events) | Exposed persons | | | | | | | | | | | |
| --- | --- | --- | --- | --- | --- | --- | --- | --- | --- | --- | --- | --- |
|  | Women | | | | Men | | | | Non-binary | | | |
|  | No | | Yes | | No | | Yes | | No | | Yes | |
|  | N | % | N | % | N | % | N | % | N | % | N | % |
| Men only | 1441 | 95.9 | 61 | 4.1 | 87 | 97.8 | 2 | 2.2 | 14 | 93.3 | 1 | 6.7 |
| Women only | 28 | 93.3 | 2 | 6.7 | 225 | 98.3 | 4 | 1.7 | 1 | 100.0 | 0 | 0 |
| Non-binary only | 1 | 25.0 | 3 | 75.0 | 2 | 100.0 | 0 | 0 | - | - | - | - |
| Unknown only | 8 | 100.0 | 0 | 0 | 19 | 95.0 | 1 | 5.0 | 1 | 100.0 | 0 | 0 |
| Men + Women | 36 | 97.3 | 1 | 2.7 | 36 | 94.7 | 2 | 5.3 | - | - | - | - |
| Men + Non-binary | 1 | 100.0 | 0 | 0 | - | - | - | - | - | - | - | - |
| Men + Unknown | 4 | 100.0 | 0 | 0 | - | - | - | - | 1 | 100.0 | 0 | 0 |
| Men + Women + Unknown | - | - | - | - | 1 | 100.0 | 0 | 0 | - | - | - | - |
| Men + Women + Non-binary + Unknown | - | - | - | - | - | - | - | - | 1 | 50.0 | 1 | 50.0 |
| Women + Non-binary | - | - | - | - | 2 | 100.0 | 0 | 0 | - | - | - | - |
| Women + Unknown | 1 | 100.0 | 0 | 0 | - | - | - | - | - | - | - | - |
| *Sum* | *1520* |  | *67* |  | *372* |  | *9* |  | *18* |  | *2* |  |

Table II E. University staff and PhD students.
N=410; Women and men who had been exposed by men only or by women only

| Gender of perpetrator or perpetrators  (in one or several  SH events) | Exposed persons | | | | | | | | | |
| --- | --- | --- | --- | --- | --- | --- | --- | --- | --- | --- |
|  | Women | | | | | Men | | | | |
|  | Disclosed | | | | | | | | | |
|  | No | | Yes | |  | No | | Yes | |  |
|  | N | % | N | % | Pearson chi square | N | % | N | % | Pearson chi square |
| Men only | 208 | 59.8 | 140 | 40.2 |  | 5 | 62.5 | 3 | 37.6 |  |
| Women only | 2 | 50.0 | 2 | 50.0 | 0.69 | 30 | 60.0 | 20 | 40.0 | 0.89 |
|  |  |  |  |  |  |  |  |  |  |  |
|  | Reported | | | | | | | | | |
|  | No | | Yes | |  | No | | Yes | |  |
|  | N | % | N | % | Pearson chi square | N | % | N | % | Pearson chi square |
| Men only | 291 | 93.6 | 57 | 16.4 |  | 7 | 87.5 | 1 | 12.5 |  |
| Women only | 2 | 50.0 | 2 | 50.0 | 0.07 | 40 | 80.0 | 10 | 20.0 | 0.62 |

Table II F. Students.
N=1850; Women and men who had been exposed by men only or by women only

| Gender of perpetrator or perpetrators  (in one or several  SH events) | Exposed persons | | | | | | | | | |
| --- | --- | --- | --- | --- | --- | --- | --- | --- | --- | --- |
|  | Women | | | | | Men | | | | |
|  | Disclosed | | | | | | | | | |
|  | No | | Yes | |  | No | | Yes | |  |
|  | N | % | N | % | Pearson chi square | N | % | N | % | Pearson chi square |
| Men only | 1325 | 88.2 | 177 | 11.8 |  | 80 | 89.9 | 9 | 10.1 |  |
| Women only | 28 | 93.3 | 2 | 6.7 | 0.39 | 214 | 93.4 | 15 | 6.6 | 0.28 |
|  | | | | | | | | | | |
|  | Reported | | | | | | | | | |
|  | No | | Yes | |  | No | | Yes | |  |
|  | N | % | N | % | Pearson chi square | N | % | N | % | Pearson chi square |
| Men only | 1441 | 95.9 | 61 | 4.1 |  | 87 | 97.8 | 2 | 2.2 |  |
| Women only | 28 | 93.3 | 2 | 6.7 | 0.48 | 225 | 98.3 | 4 | 1.7 | 0.77 |
